# Inflammaging and osteoarthritis - therapeutic opportunities

**DOI:** 10.3389/fimmu.2026.1850495

**Published:** 2026-06-23

**Authors:** Megan Chan, Phoebe Low, Shi Chee Ong, Sze Mun Choy, Wen Xing Lee, Kah Yong Goh, Kanya Moolsuwan Ng, Kimberly Chua, Jia Ying Lee, Alvin Kunyao Guo, Jiang Jia, Eng King Tan, Hong-Wen Tang, Kenon Chua

**Affiliations:** 1 Department of Orthopaedic Surgery, Singapore General Hospital, Singapore, Singapore; 2 Program in Cancer and Stem Cell Biology, Duke-National University of Singapore (NUS) Medical School, Singapore, Singapore; 3 Department of Orthopaedic Surgery, Shanghai Sixth People’s Hospital, Shanghai, China; 4 Department of Neurology, National Neuroscience Institute, Singapore, Singapore

**Keywords:** cartilage degradation, cellular senescence, chronic inflammation, CRISPR/Cas9, microRNA modulation, mTOR pathway, NF-κB signaling, osteoarthritis

## Abstract

As patients age, cellular senescence results in senescence-associated secretory phenotype (SASP). The SASP results in secretion of pro-inflammatory cytokines, chemokines, and proteases that drive chronic inflammation and cartilage degradation in osteoarthritis (OA). Accumulation of senescent chondrocytes and macrophages activates key signaling pathways such as NF-κB, MAPK/p38, mTOR, AMPK, and JAK/STAT, leading to sustained inflammation and extracellular matrix (ECM) breakdown. Recent studies have identified pharmacological and gene-based approaches that modulate SASP-related pathways. Small molecules such as anakinra, metformin, and rapamycin show potential in reducing inflammation and preserving cartilage, while microRNA modulation and CRISPR/Cas9 editing provide emerging means to regulate SASP factors at the transcriptional level. This review summarizes the molecular mechanisms by which the SASP contributes to OA pathogenesis and highlights novel therapeutic strategies aimed at attenuating inflammation, maintaining cartilage integrity, and mitigating disease progression.

## 1 Introduction

Osteoarthritis (OA) is a heterogenous degenerative disease characterized by synovitis, cartilage degeneration and osteophyte formation (1). While mechanical stress is a primary driver of OA, chronic inflammation and imbalanced macrophage polarization, together with senescent and metabolically unstable chondrocytes, further exacerbate disease progression (2). Articular cartilage integrity critically depends on the maintenance of extracellular matrix (ECM) homeostasis, as the ECM provides both structural support and biomechanical resilience. Disruption of the balance between ECM synthesis and degradation—driven by dysfunctional chondrocytes—leads to progressive cartilage breakdown and underlies OA pathogenesis (3).

Senescence, a hallmark of ageing, is a complex stress response characterized by irreversible cell-cycle arrest and the release of pro-inflammatory mediators into the surrounding microenvironment (4). This senescence-associated secretory phenotype (SASP) (4) contributes to decreased cellular proliferation, impaired tissue regeneration, and functional decline through the secretion of pro-inflammatory cytokines, chemokines, and matrix-degrading enzymes, which reinforce local inflammation, disrupt tissue homeostasis, and promote extracellular matrix breakdown as senescent cells accumulate with age (4). DNA damage, oxidative stress, and oncogenic signaling also contributes to replicative exhaustion, independent of chronological ageing (4) with the most potent being persistent DNA damage as it suppresses expression of the histone demethylase G9a, resulting in a more open chromatin state that promotes SASP-related gene transcription. Because the SASP is enriched with pro-inflammatory cytokines (e.g. CXCL1, CXCL3), cytokines (e.g. IL-1, IL-6, TNF), proteases (e.g. MMPs, ADAMTS), and growth factors (e.g. VEGF), it is thought to drive the chronic inflammation implicated in the pathogenesis of age-related diseases such as OA (4, 5). These factors act in both an autocrine manner—reinforcing the senescent phenotype within the same cell—and a paracrine manner, inducing senescence in surrounding cells. In young healthy tissues, the SASP is typically transient and maintains tissue homeostasis (6). In contrast, accumulation of SASP leads to low-level chronic inflammation commonly observed in ageing tissues.

This review aims to summarize the roles, mechanisms, and therapeutic potential of targeting inflammatory factors—particularly the senescence-associated secretory phenotype (SASP), comprising cytokines and proteases and related signaling pathways, with a focus on strategies to suppress inflammation and attenuate OA pathogenesis. 

## 2 SASP-driven inflammatory signaling in OA

Pro-inflammatory cytokines play a central role in shaping the inflammatory microenvironment in osteoarthritis (OA), driving disease progression by disrupting the balance between anabolic and catabolic processes that maintain joint tissue homeostasis (7). In OA, a sustained inflammatory milieu promotes chondrocyte dysfunction and shifts cartilage metabolism toward degradation (7).

**Figure 1:**
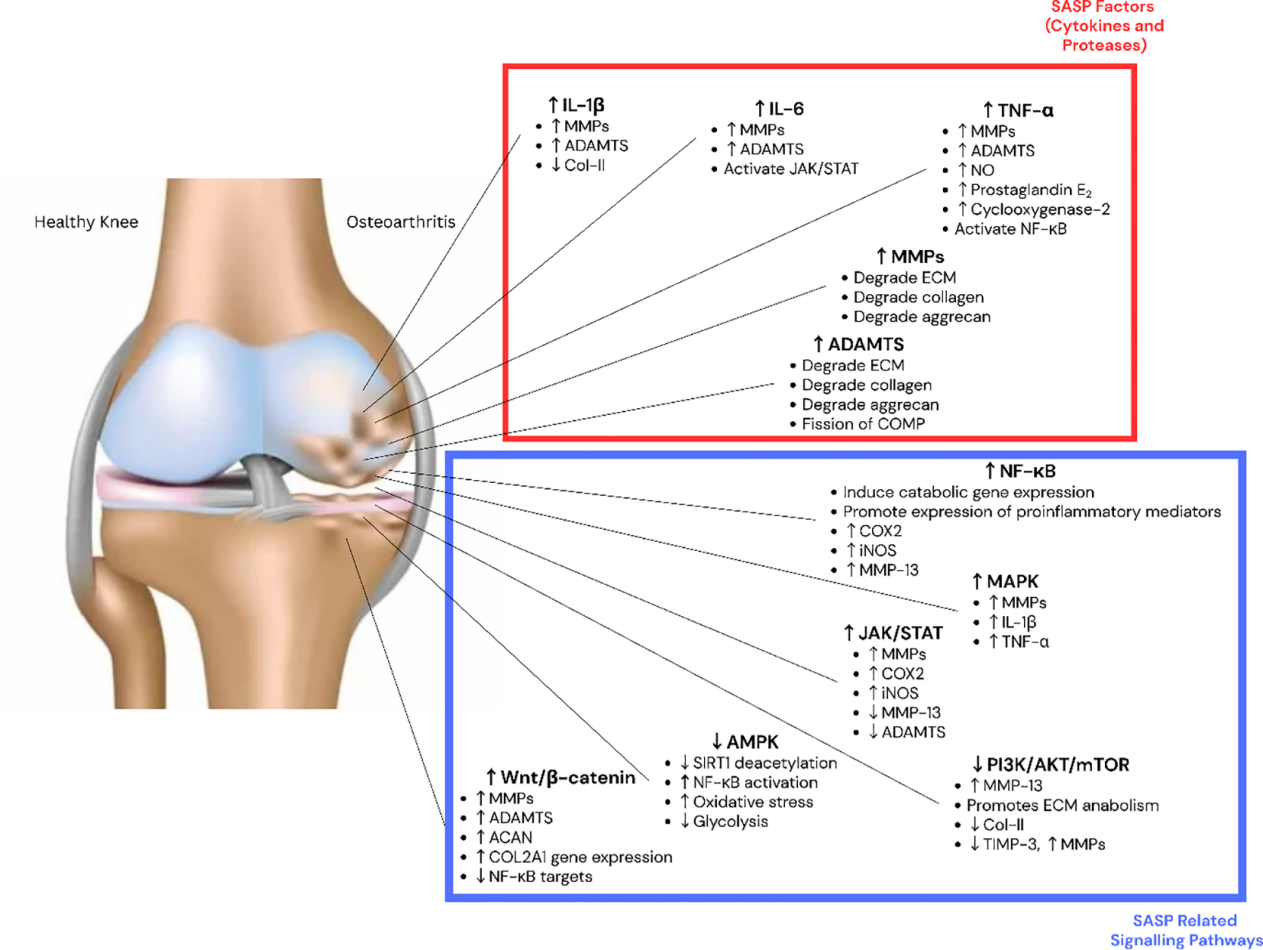
The overview of SASP factors and SASP related signaling pathways that contribute to OA. Created with BioRender.com (2026).

Among these cytokines, IL-1β, TNF, and IL-6 are key mediators of metabolic imbalance and catabolic activation in joint tissues. Interleukin-1 (IL-1) in its two forms interleukin-1α (IL-1α) and interleukin-1β (IL-1β), regulates inflammation by controlling a range of innate immune processes. IL-1β is able to initiate signal transduction pathways, increasing cartilage catabolism and suppressing cartilage anabolism (8). Tumor necrosis factor α and β (TNF-α, TNF-β), participates in various cellular events such as cell survival, proliferation, differentiation, and death by activating several signaling pathways such as nuclear factor κB (NF- κB), and c-Jun N-terminal kinase (JNK) (9). TNF is capable of inducing inflammatory gene expression and necrotic or apoptotic cell death (9). Interleukin-6 (IL-6) is a pro-inflammatory cytokine that drives the production of most acute phase proteins, which reflect the presence and severity of inflammation. IL-6 signals through its membrane-bound receptor (mIL-6R) and the co-receptor glycoprotein 130 (gp130), together initiating downstream cellular pathways (10) (**Figure 1**).

Such pro-inflammatory cytokines primarily act on chondrocytes, the resident cells of articular cartilage, and also influence synovial fibroblasts and infiltrating immune cells. They upregulate the production of proteases such as matrix metalloproteinases (MMPs) and A disintegrin and metalloproteinase with thrombospondin motifs (ADAMTS), leading to the loss of cartilage integrity and the progression of OA. MMP-1 and MMP-3 are rate-limiting enzymes in collagen degradation, while other MMPs (e.g., MMP-2, MMP-3, MMP-9) primarily target non-collagenous matrix components (11). Beyond matrix degradation, MMPs modulate cellular processes including proliferation, migration, differentiation, and apoptosis, linking extracellular matrix remodeling to both normal and pathological inflammatory responses (11). ADAMTS-1, -4, -5, -8, and -15 cleave aggrecan, making articular cartilage more susceptible to protease activity and mechanical stress, thereby accelerating cartilage degeneration. ADAMTS-7 and -12 contribute to OA progression by degrading cartilage oligomeric protein (COMP), a key regulator of cartilage ECM assembly and a potential biomarker for cartilage degeneration (12). Together, these enzymes demonstrate how inflammatory signaling can coordinate both cell behavior and matrix breakdown, highlighting potential targets for therapeutic intervention.

The SASP is related to several signaling pathways, including the NF-κB pathway, p38 mitogen-activated protein kinase (MAPK/p38) pathway, mammalian target of rapamycin (mTOR) pathway, AMP-activated protein kinase (AMPK) pathway, and Janus kinase/signal transducers and activators of transcription (JAK/STAT) pathway (13). The NF-κB, MAPK/p38, and JAK/STAT pathway are the three main pathways that play key roles in inflammation, where the dysregulation of one or more of these pathways can contribute to inflammation related diseases such as OA (**Table 1**).

**Table 1:** Therapeutic molecules targeting SASP related signaling pathways.

Molecule	Target	Effect	Clinical trial for OA
Rapamycin	mTOR	• Suppress IL-1 expression. • Decrease NF- κB activity. • Decrease IL-1α levels. • Inhibit production of SASP factors (e.g. IL-6, IL-8, CSF-2, CCL-8, BMP-4).	NA
Metformin	AMPK	• Decrease expression of MMP-3, MMP-13. • Increase expression of Col-II. • Decrease levels of SASP factors (e.g. IL1-β, IL-8, IL-6, CXCL-5). • Stimulates SIRT3-PINK1-PRKN pathway.	Yes ACTRN12621000710820
	mTOR		
TAK-242	NF-κB	• Suppress pro-inflammatory cytokines (IL-1β, IL-6, TNF-α, IL-8). • Inhibit MMP-1, VEGF. • Inhibit IκBα phosphorylation.	NA
	TLR4		
Tofacitinib	JAK/STAT	• Reduce IL-6 secretion. • Increase miR-149-5p levels. • Decrease expression of MMP-3, MMP-13.	NA (Yes for RA, ankylosing spondylitis)

### 2.1 NF-κB pathway

NF-κB is an inducible transcription factor with a primary role in immune responses, inflammatory responses, cellular differentiation, and cell survival (14). Articular chondrocytes express mechanoreceptors, cytokine receptors, TNF-R, and TL-R on their surface (15). The activation of these receptors by fibronectin fragments, mechanical stress, ageing factors or pro-inflammatory mediators, including TNF-α and IL-1β, induces NF-κB signaling pathway (15). The NF-κB pathway attenuates anabolic chondrocyte activity and induces the secretion of various matrix-degrading proteinases (15).

### 2.2 MAPK/p38 pathway

The MAPK/p38 pathway is a serine/threonine kinase signaling cascade widely present in eukaryotic cells (16). Aging-related cellular changes, including oxidative stress and senescence, can drive dysregulation of the MAPK/p38 pathway (17). This dysregulation in turn promotes exaggerated inflammatory responses and induces the expression of cartilage matrix–degrading enzymes, thereby contributing to cartilage degeneration (16).

### 2.3 JAK/STAT pathway

The JAK/STAT pathway is activated by numerous cytokines, interferons, and growth factors, and regulates key physiological processes including cell proliferation, differentiation, immune responses, and apoptosis (18). Under both aging-related conditions and pathological states such as OA, chronic exposure to pro-inflammatory cytokines leads to sustained activation of the JAK/STAT pathway. This persistent activation contributes to cartilage degradation, subchondral bone remodeling, and synovial inflammation, thereby driving disease progression (18).

### 2.4 AMPK pathway

AMPK, a serine/threonine-protein kinase, is a highly conserved energy status sensor that reacts to cellular ATP level changes in eukaryotic cells where aberrant activity is associated with mitochondrial dysfunction (19). This metabolically shifts chondrocytes from oxidative phosphorylation to glycolysis producing inflammatory mediators and catabolic enzymes, exacerbating cartilage degradation in OA (19). AMPK inhibits NF-κB activation via SIRT1 deacetylation of the p65 subunit, reducing oxidative stress in articular cartilage (19). Overall, AMPK activation helps mitigate oxidative stress, suppress inflammatory mediators, and limit catabolic enzyme production, thereby protecting cartilage integrity.

### 2.5 mTOR pathway

mTOR is a nutrient-sensing signaling cascade regulating cell growth and metabolism (20–22), with recent studies demonstrating its importance in chondrocyte metabolism, cartilage homeostasis, and OA pathophysiology (23). The PI3K/AKT/mTOR signaling pathway has been shown to be essential in maintaining joint health and is implicated in OA pathogenesis (24). Inhibition of PI3K/AKT/mTOR reduces joint damage resulting from OA by restoring cartilage homeostasis, enhancing autophagy, and suppressing inflammatory response (25). However, the PI3K/AKT/mTOR activation promotes chondrocyte proliferation and decreases apoptosis.

### 2.6 Wnt/β-catenin pathway

The SASP is related to several signaling pathways, including the Wnt/β-catenin (canonical) pathway, which regulates the transcriptional co-activator β-catenin and controls key developmental gene expression programs. Dysregulated activation of Wnt/β-catenin signaling contributes to OA by increasing inflammatory mediators, aggravating cartilage damage, and promoting synovial inflammation (26). At the same time, this pathway plays a crucial role in bone homeostasis: it promotes osteoblast differentiation and bone formation while indirectly inhibiting osteoclastogenesis (26). Therefore, the balance of Wnt/β-catenin activity is critical, as excessive or insufficient activation can simultaneously influence inflammation, cartilage integrity, and bone remodeling, highlighting its potential as a therapeutic target.

## 3 Targeting SASP factors (cytokines and proteases) involved in knee OA pathogenesis

**Figure 2:**
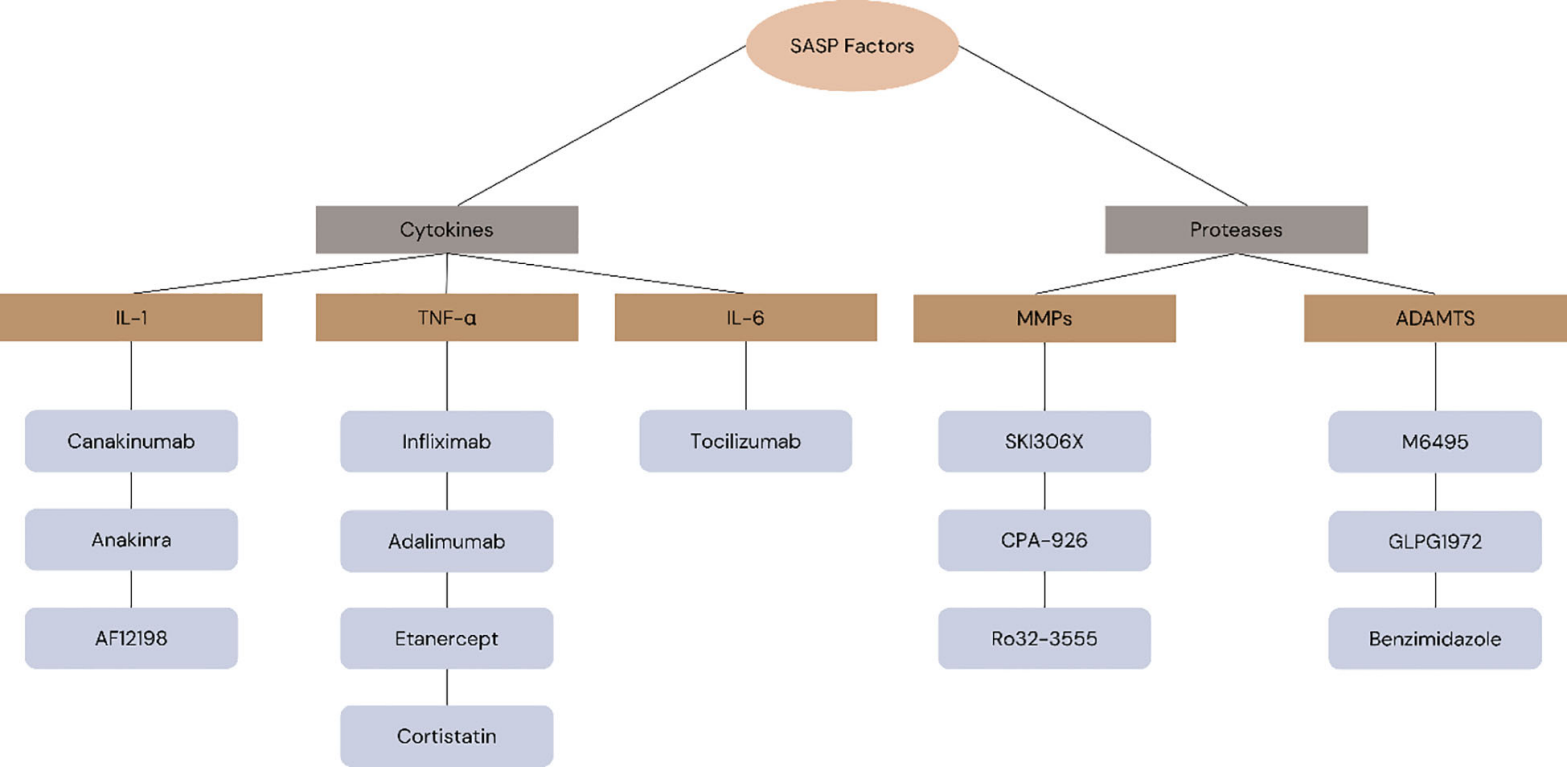
Therapeutic molecules targeting SASP factors.

The SASP drives knee osteoarthritis (OA) by releasing pro-inflammatory cytokines, chemokines, and matrix-degrading enzymes, which promote cartilage degeneration and synovial inflammation (**Figure 2**). Key mediators include cytokines such as IL-1β, TNF-α, and IL-6, and proteases including MMPs and ADAMTS, making them attractive targets for pharmacological intervention through cytokine neutralization, protease inhibition, or gene expression modulation.

Current OA treatments primarily focus on symptom management—pain relief with NSAIDs, corticosteroid injections, and physical therapy—but do not address the underlying cartilage damage or inflammatory processes (27). The lack of approved disease-modifying therapies underscores the urgent need for interventions targeting SASP-driven mechanisms, prompting growing interest in drugs that directly neutralize pro-inflammatory cytokines and inhibit matrix-degrading proteases (**Figure 2**).

### 3.1 Cytokines

#### 3.1.1 IL-1β

The interleukin-1 family (IL-1F) comprises 11 members, with the pro-inflammatory cytokine IL-1β being a well-known catabolic factor (28). IL-1β is produced by chondrocytes, mononuclear cells, osteoblasts and synovial cells (29), and binds to IL-1 type 1 receptor (IL-1R1) and IL-1 type 2 receptor (IL-1R2), a decoy receptor. IL-1β is a key factor in the pathogenesis of OA via the activation of several signaling pathways such as nuclear factor κB (NF-κB) and MAPK (30). IL-1β is one of the key factors implicated in cartilage degradation, capable of downregulating type II collagen (Col-II) and proteoglycan (PG) synthesis, stimulating the release of MMPs and ADAMTS. The action of IL-1β triggers the catabolism of cartilage by MMPs, cleaving most of the components of the ECM (31). Chondrocytes not only express IL-1R1, but upregulate IL-1R1 levels in patients suffering from OA, with OA chondrocytes displaying increased sensitivity to IL-1β, and higher levels of IL-1β binding compared to normal chondrocytes (32).

Canakinumab is a human anti-IL-1β monoclonal antibody (mAb) developed by Novartis and approved for the treatment of cryopyrin-associated periodic syndromes (CAPS) and Still’s disease. It binds specifically to IL-1β, preventing its interaction with the IL-1 receptor (IL-1R1) and thereby inhibiting downstream inflammatory signaling. Exploratory analyses of OA patients suggest that canakinumab treatment may reduce the incidence of hip and knee replacements, with rates of 0.31 and 0.54, respectively, compared to placebo, indicating a potential effect on disease progression (29).

*In vitro* studies using human OA chondrocytes show that canakinumab not only restores anabolic activity but also reduces cartilage degradation induced by IL-1β and TNF-α. The suppression of TNF-α–mediated effects is likely indirect: IL-1β blockade can attenuate NF-κB and other pro-inflammatory signaling pathways that are shared between IL-1β and TNF-α, leading to decreased nitric oxide (NO) production and preservation of proteoglycan (PG) content (33). These results highlight the potential of canakinumab to modulate key inflammatory networks in OA and support further clinical evaluation as a disease-modifying therapy.

Anakinra is a recombinant, non-glycosylated form of human IL-1 receptor antagonist (IL-1Ra) and is approved for the treatment of rheumatoid arthritis (RA). Human IL-1Ra naturally binds to the IL-1 receptor (IL-1R1), blocking IL-1β from activating the receptor, whereas anti-IL-1β antibodies directly neutralize IL-1β. By preventing IL-1β signaling, anakinra reduces joint inflammation and slows cartilage erosion. In a canine osteoarthritis model with anterior cruciate ligament transection, intra-articular injections of recombinant human IL-1Ra (rHuIL-1Ra) decreased both the incidence and size of osteophytes and reduced macroscopic and microscopic cartilage lesions (34). However, a randomized, double-blind, placebo-controlled clinical study reported that intra-articular injections of anakinra at doses of 50 mg or 150 mg did not significantly improve OA symptoms compared with placebo (35). These findings indicate that further research is needed to optimize IL-1Ra formulation and administration to maximize therapeutic efficacy in OA.

AF12198 is a recombinant peptide derived from a family of peptide antagonists, being the first low molecular weight IL-1Ra to display *in vivo* activity (36). It binds specifically to IL-1R1 blocking IL-1-induced activities both *in vitro* and *in vivo* (36). In Cynomolgus monkeys, AF12198 infusion decreased IL-1 induction of IL-6 in four of the six subjects, but AF-12198 was quickly degraded by endopeptidases activity to two non-active metabolites (36). Therefore, it was shown that AF-12198 is able to block IL-1 induced activities, however, direct therapeutic usage of the peptide may be limited due to rapid metabolism and unfavorable pharmacokinetics (36).

#### 3.1.2 TNF-α

**TNF-α** is an inflammatory cytokine that plays a key role in the inflammation process and cartilage degradation in OA pathogenesis (37). It signals through two cell-surface receptors, TNFR1 and TNFR2, which are broadly expressed across human tissues, including articular chondrocytes (38). TNF-α upregulates the expression of matrix metalloproteinases (MMPs) and aggrecanases (ADAMTS), accelerating extracellular matrix (ECM) degradation, including the loss of type II collagen (Col-II) and aggrecan, thereby promoting OA progression (39, 40). In addition, TNF-α induces a broad inflammatory response characterized by increased production of nitric oxide (NO), inducible nitric oxide synthase (iNOS), cyclooxygenase-2 (COX-2), and prostaglandin E2 (PGE2) (41, 42). Among these, activation of the COX-2/PGE2 axis represents a major downstream pathway through which TNF-α amplifies inflammation, pain sensitization, and cartilage catabolism (43). PGE2 further reinforces disease progression by enhancing catabolic enzyme expression and sustaining NF-κB activation, thereby establishing a feed-forward inflammatory loop that exacerbates joint degeneration (44). Emerging evidence also suggests that prostaglandin signaling may interact with senescence-associated secretory phenotype (SASP) factors, linking chronic inflammation to cellular senescence in OA (45). Although pharmacological inhibition of this pathway using nonsteroidal anti-inflammatory drugs (NSAIDs) and selective COX-2 inhibitors remains a cornerstone of symptomatic OA treatment, their long-term disease-modifying efficacy is limited (46). Beyond cartilage, TNF-α also contributes to osteoclast activation and subchondral bone resorption. Elevated TNF-α levels have been detected in synovial fluid, synovial membrane, subchondral bone, and cartilage in OA patients. Notably, TNF-α levels within circulating extracellular vesicles (EVs), membrane-bound particles released by cells that transport proteins, lipids, and nucleic acids between tissues (47, 48), are significantly increased in patients with progressive OA. Baseline EV-associated TNF-α levels are also associated with predicted knee OA progression (49).

Infliximab is a purified, recombinant DNA-derived chimeric IgG monoclonal antibody that targets TNF-α and is widely used for inflammatory diseases such as Crohn’s disease, rheumatoid arthritis, ankylosing spondylitis, psoriatic arthritis, and plaque psoriasis. In addition to neutralizing soluble TNF-α, infliximab binds transmembrane TNF-α, inducing complement fixation and antibody-dependent cytotoxicity. In a clinical study of erosive hand OA, patients received intra-articular infliximab injections and were monitored over 12 months; all participants experienced symptomatic relief, with improved WOMAC scores at 6 and 12 months, and a trend toward reduced radiographic progression, although this did not reach statistical significance (50). These findings provide preliminary evidence that infliximab may attenuate OA pathogenesis and warrant further investigation in well-designed trials.

Adalimumab is a fully human recombinant monoclonal antibody that targets TNF-α, blocking its interaction with the p55 and p75 TNF cell surface receptors. It is highly effective in treating adults with active rheumatoid arthritis who have had an insufficient response to DMARDs. In the context of OA, however, clinical evidence remains mixed. One randomized controlled trial comparing hyaluronic acid with intra-articular adalimumab reported improvements in patients with moderate to severe knee OA (51), whereas a randomized, double-blind, placebo-controlled crossover trial in erosive hand OA found no significant differences in primary or secondary outcomes at 12 weeks (52).

These divergent findings may reflect heterogeneity in OA subtypes, disease severity, joint location, dosing regimen, treatment duration, and the relative contribution of TNF-α–driven inflammation across patient populations. Overall, while TNF-α inhibition with adalimumab may show potential benefit in selected OA subgroups, the current clinical evidence remains inconsistent. Further well-designed, adequately powered clinical trials are required to clarify its therapeutic efficacy and define its role in OA management.

Etanercept is a TNF-α antagonist that functions as a soluble receptor construct, consisting of two p75 TNF receptors fused to the Fc portion of human IgG. It is widely used to manage autoimmune disorders, including plaque psoriasis, rheumatoid arthritis, psoriatic arthritis, juvenile idiopathic arthritis, and ankylosing spondylitis. Intra-articular injection of etanercept for knee OA has been reported to provide significant pain relief at 1 and 2 weeks according to VAS scores, and at 4 weeks according to WOMAC scores (53). However, another study found no significant pain reduction after 24 weeks, with mean VAS differences compared to placebo being statistically insignificant (54). These discrepancies likely reflect heterogeneity in OA phenotypes, disease stage and severity, pharmacokinetic and residence time of intra-articular etanercept, dosing strategies, and inter-individual variability in TNF-α–driven inflammatory activity. In addition, OA is a multifactorial disease in which TNF-α represents only one of several contributing pathways to pain and tissue degeneration. Together, these findings suggest that while TNF-α blockade may provide transient benefit in selected contexts, its overall therapeutic efficacy in OA remains variable, underscoring the need for further studies to define optimal treatment strategies and identify responsive patient subgroups.

Cortistatin is a cyclic neuropeptide that binds to somatostatin receptors and has been shown to interact with TNF receptors (TNFRs) *in vitro* (37). By competing with TNF-α for TNFR binding, cortistatin suppresses the pro-inflammatory and catabolic actions of TNF-α on chondrocytes, which are key drivers of cartilage degradation in OA. *In vivo* studies further support its protective role, demonstrating that cortistatin reduces chondrocyte apoptosis and attenuates Wnt/β-catenin signaling, a pathway implicated in OA-related cartilage degeneration and osteophyte formation (37). These effects collectively suggest that cortistatin can modulate multiple aspects of OA pathology: it dampens inflammatory signaling, limits cartilage breakdown, and reduces aberrant chondrocyte activity. By targeting both TNF-α–mediated catabolism and key signaling pathways involved in cartilage homeostasis, cortistatin emerges as a potential multifaceted therapeutic agent for slowing OA progression.

#### 3.1.3 IL-6

IL-6 is a pro-inflammatory cytokine with pleiotropic effects on inflammation, immune response, and hematopoiesis. It signals through either the membrane-bound IL-6 receptor (mIL-6R) or the soluble IL-6 receptor (sIL-6R) (55). Binding of IL-6 to these receptors triggers downstream signaling, predominantly via the JAK/STAT pathway, which is further regulated by glycoprotein 130 (gp130) forming a complex with IL-6/mIL-6R (55). In chondrocytes, IL-6 induces the expression of metalloproteinases and aggrecanases, contributing to cartilage degradation. Elevated IL-6 levels in synovial fluid have been correlated with OA severity and progression. Experimental studies using the destabilization of the medial meniscus (DMM) murine model—a well-established surgical model that mimics post-traumatic OA—have shown that systemic IL-6 blockade attenuates cartilage lesions, reduces osteophyte formation, and limits synovial inflammation (56). These findings suggest that IL-6 is a key mediator of cartilage breakdown and joint pathology, highlighting its potential as a therapeutic target in OA.

Tocilizumab is a genetically-engineered, humanized anti-interleukin-6 receptor (IL-6R) monoclonal antibody of the immunoglobulin G1_k_ subclass, principally used in the treatment of RA, systemic juvenile idiopathic arthritis. It inhibits binding of IL-6 to its receptors, effectively reducing its pro-inflammatory effects by competing with both the membrane-bound and soluble forms of IL-6R. However, *in vitro* studies have reported that tocilizumab does not inhibit on TNF-α, IL-1β, IL-15, or IL-2. *In vitro* and *in vivo* studies with regards to cynomolgus monkeys with collagen-induced arthritis revealed that tocilizumab was able to inhibit inflammation both locally and systemically in joints. It was also observed that IL-6 inhibition by tocilizumab results in the normalization of the inflammation driven osteoclastic bone destruction, with normal bone homeostasis returning with continuous IL-6 inhibition by tocilizumab. However, another study concluded that tocilizumab was no more effective than placebo with regards to pain relief (95). These findings suggest that the role of IL-6 signaling as a therapeutic target in OA warrants further clarification.

### 3.2 Proteases

#### 3.2.1 MMPs

MMPs are a family of zinc-dependent endopeptidases involved in ECM remodeling (57). They are secreted as inactive proenzymes and activated at the tissue level through N-terminal cleavage by other proteases (57). MMP overexpression is observed in pathological conditions such as OA, where dysregulated MMP activity leads to aberrant ECM degradation and disease progression. MMP activity is regulated by endogenous tissue inhibitors of MMPs (TIMPs) (57). Based on substrate specificity and cellular localization, MMPs are classified into membrane-type MMPs, collagenases, gelatinases, stromelysins, and matrilysins (57). Among these, MMP-13 is highly expressed in OA chondrocytes and plays a central role in type II collagen degradation in cartilage (57).

SKI306X (JOINS, SK Chemicals) is a fractionated extract composed of three traditional oriental herbal ingredients, *Clematis mandshurica*, *Trichosanthes kirilowii*, and *Prunella vulgaris*. It is clinically approved for the treatment of OA in South Korea, previously found to possess anti-inflammatory, analgesic, and cartilage protective effects. In a double-blind placebo-controlled study investigating the effects of SKI306X in knee OA patients (58), improvement was observed in every efficacy parameter in the SKI306X-treated groups as compared to the placebo group after 4 weeks (58). Mechanistically, SKI306X suppresses the degradation of proteoglycans (PGs) in a concentration-dependent manner in rabbit explant cultures, counteracting OA-like changes induced by intra-articular collagenase (59). Proteoglycans are critical components of the cartilage extracellular matrix (ECM), providing compressive strength and maintaining tissue hydration; thus, preventing PG degradation directly contributes to cartilage preservation and mechanical resilience. In addition, SKI306X inhibits the expression of MMP-3 and MMP-13, further preventing collagen breakdown (59). Together, these effects highlight SKI306X as a promising therapeutic candidate for both slowing cartilage degradation and managing OA symptoms.

CPA-926 is a pro-drug of esculetin, a compound which inhibits inflammatory pathways (60). *Yamada* et al. demonstrated that esculetin inhibited matrix degradation in rabbit cartilage explants by suppressing IL-1α–induced proteoglycan release in a dose-dependent manner, accompanied by reduced levels of proMMP-1 and proMMP-3 on Western blot analysis. *In vivo*, rabbits treated with CPA-926 at 400 mg/kg showed a reduced erosive area on the tibial plateau compared with controls. Similarly, *Watanabe* et al. concluded that esculetin effectively inhibits IL-1α-induced PG release and transcriptionally down-regulates proMMP-1 and proMMP-3 production, suppressing PG reduction in rabbit chondrocytes (60). CPA-926 may be a potential therapeutic strategy against OA.

Ro32–3555 is an orally active inhibitor of collagenases, specifically MMP-3, MMP-2, and MMP-9 (61). It was designed to balance favorable bioavailability with selective collagenase inhibition and has been shown to prevent cartilage degradation both *in vitro* and *in vivo* (61)*. Lewis* et al. showed that Ro32–3555 protects cartilage by inhibiting MMPs responsible for type II collagen (Col-II) degradation. Specifically, Ro32–3555 prevented IL-1α-induced breakdown of bovine nasal cartilage explants by suppressing these collagen-degrading enzymes (61). Consistent with this mechanism, Ro32–3555 also demonstrated protective effects on articular cartilage in Propionibacterium acnes-induced rat monoarthritic models, likely by preserving extracellular matrix integrity and limiting inflammation-driven cartilage damage (61). Similarly, *Brewster* et al., demonstrated that Ro32–3555 attenuated structural damage in STR/ORT mouse models of OA (62). Significant reduction of joint space narrowing and osteophyte formation was reported when treated with 10–50 mg/kg of Ro32-3555, providing evidence that Ro32–3555 may be able to protect articular cartilage from degeneration (62). These data suggest that Ro32–3555 as a plausible therapeutic opportunity in mitigating OA progression through targeting MMPs.

#### 3.2.2 ADAMTS

The ADAMTS protease family is categorized into different groups, aggrecanase, procollagen N-peptidase, COMP enzyme, von-Willebrand factor proteolytic enzyme, and orphan enzymes. These functions are tightly associated with the ECM and cartilage degradation found in OA, suggesting that ADAMTS are a potential therapeutic target. ADAMTS have been reported to regulate the release and expression levels of inflammatory mediators by regulating relevant signaling pathways (63). A number of ADAMTS have been associated with cartilage breakdown prevalent in OA, among which, ADAMTS-4 and ADAMTS-5 (classified as aggrecanases), have been extensively studied (64). ADAMTS-4 is induced by pro-inflammatory mediators while ADAMTS-5 is expressed in human cartilage (64). Interestingly, *Adamts5*-deleted mice, but not *Adamts*4-deleted mice, were exhibited to have significantly reduced cartilage degradation in OA and inflammatory arthritis models, giving significance to ADMTS-5 (65).

M6495 is a 28.1 kDa bivalent and bifunctional Nanobody, composed of two sequence-optimized variable domains derived from heavy-chain-only llama antibodies. It exhibits high specificity for ADAMTS-5, a key aggrecanase, without binding to ADAMTS-1, ADAMTS-4, or ADAMTS-15 (66). Aggrecan and type II collagen (Col-II) are essential components of the cartilage extracellular matrix (ECM), providing compressive strength, elasticity, and structural integrity. Their degradation is a hallmark of OA cartilage degeneration, leading to loss of joint function, cartilage erosion, and progression of the disease. *Ex vivo* studies by Siebuhr et al. demonstrated that M6495 significantly inhibited cytokine-induced degradation of aggrecan and Col-II, without affecting Col-II production, highlighting its potential as an anti-catabolic therapy for OA (66). Additionally, two phase I double-blind, randomized, placebo-controlled studies by Bihlet et al. confirmed that M6495 was safe and well tolerated at weekly doses up to 300 mg for six weeks (66). Sharma et al. further reported that M6495 reduced the release of cartilage degradation biomarkers and aggrecan fragments, which in turn decreased activation of toll-like receptor 2 (TLR2) (67). TLR2 is an innate immune receptor expressed in chondrocytes and synovial cells that is activated by cartilage breakdown products and contributes to OA inflammation by promoting the production of pro-inflammatory cytokines and catabolic enzymes. By simultaneously preventing ECM degradation and limiting TLR2-mediated inflammatory signaling, M6495 demonstrates both chondroprotective and anti-inflammatory effects, addressing two central mechanisms of OA pathology. While these findings support its therapeutic potential, further studies are needed to evaluate long-term efficacy and clinical outcomes in OA patients.

GLPG1972 is a potent and selective ADAMTS-5 inhibitor containing a hydantoin moiety that acts as a zinc-binding group. It exhibits approximately 8-fold selectivity for ADAMTS-5 over ADAMTS-4, with even higher selectivity (ranging from 62-fold to over 5000-fold) against other metalloproteinases (68). This high degree of selectivity is important because it minimizes off-target inhibition of other metalloproteinases, which could otherwise lead to unintended tissue remodeling, toxicity, or side effects, thereby improving the safety profile of the drug. In a randomized, placebo-controlled, double-blind Phase 1b study, GLPG1972 was well tolerated and achieved a 50% reduction in cartilage breakdown biomarkers over four weeks (68). However, in a subsequent randomized, double-blind, placebo-controlled, dose-ranging Phase 2 trial (Schnitzer et al.), no significant difference in central medial femorotibial compartment (cMFTC) cartilage volume loss was observed compared with placebo (69). The divergence between early biomarker responses and later structural outcomes may reflect differences in study duration, challenges in achieving sufficient drug exposure within avascular cartilage tissue, or the multifactorial nature of osteoarthritis, in which inhibition of a single target may be insufficient to modify disease progression. Collectively, these findings indicate that while GLPG1972 demonstrates pharmacological selectivity and early biological activity, its clinical efficacy in osteoarthritis remains unconfirmed.

Benzimidazole is a heterocyclic aromatic compound known for its anti-inflammatory and analgesic properties (70). *Sogame* et al. designed a series of benzimidazole derivatives by scaffold hopping from a thiazole-based compound previously shown to inhibit ADAMTS-5 and suppress spontaneous aggrecan degradation in IL-1–stimulated bovine cartilage (71). While the thiazole compound was potent, it exhibited poor transmembrane permeability, limiting its ability to reach chondrocytes and cartilage ECM effectively. Replacing the thiazole with a benzimidazole scaffold enhanced lipophilicity and molecular stability, improving membrane permeability and tissue penetration, which is critical for targeting ADAMTS-5 in cartilage.

Among these derivatives, the compound featuring a benzimidazole fused to a thiazolidin-4-one with an n-propyl substituent emerged as the most potent, showing moderate selectivity toward MMP-2 and high selectivity against ADAMTS-4, MMP-3, MMP-14, and TNF-α–converting enzyme (TACE) (71). These improvements suggest that benzimidazole-based ADAMTS-5 inhibitors could more effectively suppress cartilage degradation in OA. Overall, benzimidazole derivatives warrant further investigation to evaluate their therapeutic potential and clinical viability in osteoarthritis.

### 3.3 Gene regulation

#### 3.3.1 MicroRNA

MicroRNA (miRNA) modulation is an emerging strategy for attenuating OA progression, given the critical roles of miRNAs in cartilage homeostasis and extracellular matrix regulation. Several miRNAs, including miR-145, miR-139, miR-9, miR-193b, and miR-320, have been implicated in OA pathogenesis and chondrocyte function (72–76).

Inhibition of miR-145 attenuated IL-1β–induced ECM degradation by restoring type II collagen, aggrecan, and glycosaminoglycan (GAG) levels, while reducing MMP-13 production (72). MiR-139 is overexpressed in damaged OA cartilage compared with smoother cartilage, suppressing the expression of monocyte chemoattractant protein-induced protein 1 (MCPIP1), a negative regulator of IL-6, thereby increasing IL-6 expression (73). Similarly, miR-9 overexpression in OA chondrocytes increased IL-6 levels and reduced MCPIP1 expression, highlighting its role in promoting local inflammatory signaling (74). Collectively, miR-9 and miR-139 contribute to OA cartilage degeneration by enhancing pro-inflammatory cytokine production, leading to ECM breakdown.

In contrast, miR-193b appears to act as a negative regulator of inflammation. Hou et al. demonstrated that miR-193b is upregulated in IL-1β–stimulated cells and suppresses TNF-α expression, suggesting a role in limiting inflammatory severity (75). *Wang* et al. similarly reported that miR-193b expression negatively correlates with inflammatory markers in both OA and systemic inflammatory conditions such as sepsis, indicating that its anti-inflammatory effects may extend beyond cartilage to broader cytokine-mediated pathologies (75). This provides a rationale for exploring miR-193b modulation as a strategy to protect cartilage integrity by restraining excessive inflammatory signaling.

MiR-320 also plays a key role in maintaining cartilage matrix homeostasis. It is expressed during late chondrogenic differentiation and directly regulates MMP-13 expression (76). In OA cartilage, miR-320 levels are significantly reduced, leading to increased MMP-13 production, decreased collagen deposition, and lower COL2A1 expression. Conversely, upregulation of miR-320 suppresses MMP-13 and enhances collagen deposition, highlighting its protective role in cartilage ECM maintenance (76). Together, these findings suggest that miRNA dysregulation contributes both to enhanced cartilage degradation and to altered inflammatory signaling in OA. Therapeutic strategies that restore protective miRNAs, such as miR-193b and miR-320, or inhibit catabolic miRNAs, such as miR-9 and miR-139, may help preserve cartilage integrity, reduce ECM breakdown, and slow OA progression.

#### 3.3.2 Gene editing modalities

CRISPR-Cas9 gene-editing technology, consisting of a guide RNA to target specific genes and the CRISPR-associated protein 9 (Cas9) endonuclease to induce double-strand DNA breaks, has emerged as a promising therapeutic strategy for OA by enabling precise modulation of disease-relevant genes and signaling pathways (77). *Karlsen* et al. used CRISPR–Cas9 to silence IL-1 receptor type 1 (IL1-R1) in human articular chondrocytes (hACs), incorporating a puromycin resistance gene to select successfully edited cells (78). Upon exposure to recombinant IL-1β, control hACs showed marked inflammatory responses, whereas IL1-R1–deficient cells exhibited minimal inflammation, demonstrating that direct gene editing can effectively block pro-inflammatory signaling in OA-relevant cells (78).

Building on this concept, Brunger et al. engineered mouse induced pluripotent stem cells (iPSCs) with a CRISPR–Cas9–mediated autoregulatory anticytokine system (79). By inserting genes encoding anti-inflammatory mediators such as IL-1Ra or soluble TNFR1 (sTNFR1) into the Ccl2 locus, these modified iPSCs could sense elevated IL-1 or TNF-α levels and autonomously produce corresponding antagonists, creating a self-regulating feedback loop that modulates inflammatory pathways in response to local cytokine concentrations (79).

Currently, the application of CRISPR in OA is largely at the preclinical stage, focused on ex vivo modification of chondrocytes or iPSCs and proof-of-concept in animal models. Nevertheless, these studies highlight the potential of CRISPR-based strategies to precisely interfere with OA-related inflammatory mechanisms, providing a foundation for the development of gene- and cell-based therapies that can attenuate cartilage degradation, restore joint homeostasis, and offer long-term disease-modifying effects.

## 4 Targeting SASP related signaling pathways

Rapamycin is an immunomodulatory macrolide antibiotic that inhibits the mammalian target of rapamycin (mTOR) signaling pathway, thereby inducing autophagy (80–84). By shifting chondrocytes from stress responses toward nutrient-survival pathways, mTOR inhibition can restore chondrocyte health, reduce senescence, and improve cartilage homeostasis (80). However, mTOR normally exerts negative feedback on the PI3K/AKT pathway, and its inhibition can inadvertently enhance PI3K/AKT/NF-κB signaling, potentially increasing the expression of matrix metalloproteinases (MMPs) and promoting extracellular matrix degradation. Despite this dual effect, experimental evidence suggests that the protective benefits predominate in OA contexts. Dhanabalan et al. demonstrated that rapamycin prevented chondrocyte senescence, induced autophagy, decreased inflammatory markers, and mitigated post-traumatic OA in mice (80). Similarly, *Caramés* et al. observed a significant reduction in cartilage degradation and synovitis in rapamycin-treated mice compared with controls (85). These findings indicate that, although mTOR inhibition can theoretically enhance MMP production, the net effect in OA models favors chondrocyte preservation, autophagy activation, and reduced joint inflammation.

Metformin is a synthetic biguanide that has been used clinically for the treatment of type II diabetes for over six decades. Beyond its glucose-lowering effects, metformin exhibits anti-inflammatory, immunomodulatory, analgesic, lipid-lowering, weight-reducing, anti-aging, and anticancer properties (86). Mechanistically, metformin inhibits complex I of the mitochondrial respiratory chain, regulating cellular energy homeostasis through activation of AMPK. AMPK, a central energy sensor, is activated via phosphorylation of its α subunit by upstream kinases such as liver kinase B1 (LKB1) and calcium/calmodulin-dependent protein kinase kinase 2 (CAMKK2). Activation of AMPK suppresses inflammatory signaling, mitigates oxidative stress, regulates autophagy, and inhibits pain signaling, suggesting that metformin may reduce chondrocyte apoptosis and OA-related pain (87). Experimental studies in chondrocytes and mouse OA models have shown that metformin suppresses the mTORC1 pathway, leading to decreased chondrocyte apoptosis and increased expression of autophagy-related genes (88). Clinically, a double-blind, placebo-controlled randomized trial by Pan et al. demonstrated that 2000 mg daily metformin reduced knee pain at 6 months (89). Similarly, Alimoradi et al. reported that metformin significantly decreased knee swelling in OA patients. Together, these findings indicate that metformin can improve key OA outcomes—pain, inflammation, and cartilage preservation—by targeting metabolic and inflammatory pathways.

TAK-242 is a selective Toll-like receptor 4 (TLR4) inhibitor that binds to the intracellular domain of TLR4, preventing its association with adaptor proteins and subsequent downstream signaling (90). *In vitro* studies using cells from OA patients demonstrated that TAK-242 effectively suppresses LPS-induced overexpression of pro-inflammatory cytokines, including IL-1β, IL-6, TNF-α, and TLR4 itself, while inhibiting IκBα phosphorylation, a key regulator of NF-κB signaling (90). These results suggest a strong anti-inflammatory potential at the cellular level. However, *in vivo* findings present a more complex picture. TAK-242 treatment in Receptors for Advanced Glycation End Products (RAGE) knockout mice paradoxically worsened OA severity (91). This discrepancy may be explained by behavioral or biomechanical effects: TLR4 inhibition likely reduces pain perception, leading to increased joint use or overloading, which in turn accelerates cartilage damage. This example underscores the importance of considering systemic and biomechanical factors in addition to molecular anti-inflammatory effects when evaluating therapeutic strategies for OA. Further studies are needed to clarify the net impact of TAK-242 on joint health *in vivo* and to identify conditions under which TLR4 inhibition may be beneficial.

Tofacitinib is an oral targeted synthetic small-molecule inhibitor of JAKs and is FDA-approved for the treatment of rheumatoid arthritis, psoriatic arthritis, ulcerative colitis, and polyarticular-course juvenile idiopathic arthritis. It preferentially inhibits JAK1 and JAK3, thereby blocking phosphorylation and activation of signal transducers and activators of transcription (STATs) involved in innate and adaptive immune signaling to reduce inflammation. Recent studies have highlighted the role of microRNAs in mediating tofacitinib’s effects in osteoarthritis. *Chiu* et al. reported that intra-articular administration of tofacitinib attenuated OA progression both *in vitro* and *in vivo* by upregulating miR-149-5p, which suppresses the expression of pro-inflammatory cytokines and downstream JAK/STAT signaling, contributing to its anti-inflammatory effect (92). Similarly, *Zhang* et al. demonstrated tofacitinib delayed ECM degradation and enhanced chondrocyte autophagy, mitigating articular cartilage degeneration *in vivo* (93). These findings suggest that miR-149-5p is a critical mediator of tofacitinib’s protective effects in OA, linking JAK/STAT inhibition to the regulation of chondrocyte inflammation and survival.

## 5 Discussion

This review examines recent advances in targeting SASP and related signaling pathways as therapeutic strategies to slow OA progression. Previous reviews have largely focused on cytokine- and senescence-targeted interventions, often treating proteases as downstream effectors of cytokine-driven inflammation rather than as independent therapeutic targets. However, accumulating evidence suggests that proteases such as MMPs and ADAMTS not only mediate ECM breakdown but can also propagate inflammatory signaling, amplify cartilage damage, and contribute to disease progression independently of cytokine activity. By highlighting proteases as key drivers of cartilage degradation, this review provides a more comprehensive perspective on OA pathogenesis and underscores the potential of directly targeting these enzymes as complementary or standalone therapeutic strategies.

Furthermore, while there has been progression in *in vivo* studies for the therapeutic strategies discussed above, further research and investigation is still required to establish their true clinical therapeutic potential. In this paper, drugs undergoing varying clinical stages were reviewed, with most having undergone *in vivo* trials, in mouse models and in cynomolgus monkeys. Additionally, canakinumab, anakinra, infliximab, adalimumab, etanercept, SKI306X, M6495, GLPG1972, metformin, and tofacitinib have undergone human clinical trials, though not all exclusively for OA.

Notably, GLPG1972, which was assessed exclusively in OA, did not demonstrate clinical efficacy beyond Phase II trials (69), whereas tofacitinib has shown therapeutic benefit in other inflammatory conditions such as rheumatoid arthritis, supporting its potential for repurposing in OA (94). This review also incorporates emerging clinical evidence, including the advancement of metformin into human clinical evaluation (ACTRN12621000710820) (89). Beyond cytokine inhibition and senescence-targeted approaches, we further highlight gene-regulatory strategies, particularly microRNA-based modulation and CRISPR–Cas gene editing, as emerging therapeutic directions. By integrating recent preclinical and clinical findings, this review provides a comprehensive overview of inflammatory and aging-related mechanisms in OA pathogenesis and outlines future avenues for therapeutic development.

## 6 Conclusion

Accumulating evidence indicates that osteoarthritis (OA) is not solely a degenerative disorder but is partly driven by chronic inflammation and cellular senescence. OA encompasses heterogeneous pathological processes, including age-related disruption of cartilage matrix and chondrocyte homeostasis, biomechanical abnormalities, and inflammation secondary to aging or injury. This review examined therapeutic strategies targeting the senescence-associated secretory phenotype (SASP), with emphasis on inhibiting SASP factors such as pro-inflammatory cytokines and proteases, as well as modulating key signaling pathways including Wnt, NF-κB, MAPK, JAK/STAT, mTOR, and AMPK.

Beyond conventional pharmacological strategies, novel approaches such as miRNA modulation and gene editing technologies (CRISPR-Cas) offer promising avenues to regulate SASP factors at the transcriptional and epigenetic levels. These strategies may overcome existing limitations associated with unfavorable pharmacokinetics such as systemic toxicity and poor bioavailability.

Collectively, these strategies mark a shift from symptom management to disease treatment in OA. Nevertheless, further studies and clinical trials are necessary to bring these findings from research into clinical practice to establish optimal treatments for OA.

## 7 Literature search

This review was prepared through a structured literature search using Pubmed. The search was performed using combinations of the following keywords: “osteoarthritis,” “senescence-associated secretory phenotype,” “SASP,” “cellular senescence,” “inflammation,” “cytokines,” “proteases,” “MMP,” “ADAMTS,” “cartilage degradation,” “NF-κB,” “mTOR pathway”, “JAK/STAT,” “microRNA,” “CRISPR,” “senolytics,” “senomorphics,” and “clinical trial.” Additional searches were performed for specific therapeutic agents discussed in this review, including canakinumab, anakinra, infliximab, adalimumab, etanercept, SKI306X, M6495, GLPG1972, metformin, and tofacitinib. Priority was given to original research articles, clinical trials, and recent review articles directly relevant to OA pathogenesis or treatment. The timeframe of the literature span from 1996 and up to 2026.
